# Magnolin promotes autophagy and cell cycle arrest via blocking LIF/Stat3/Mcl-1 axis in human colorectal cancers

**DOI:** 10.1038/s41419-018-0660-4

**Published:** 2018-06-13

**Authors:** Haiyang Yu, Shuangshuang Yin, Shiyue Zhou, Yingying Shao, Jiachen Sun, Xu Pang, Lifeng Han, Yi Zhang, Xiumei Gao, Chengyun Jin, Yuling Qiu, Tao Wang

**Affiliations:** 1 Tianjin State Key Laboratory of Modern Chinese Medicine, Tianjin University of Traditional Chinese Medicine, Tianjin, 300193 China; 2 School of Pharmaceutic al Sciences, Key Laboratory of State Ministry of Education, Key Laboratory of Henan province for Drug Quality Control and Evaluation, Zhengzhou University, Zhengzhou, Henan 450001 China; 3 School of Pharmacy, Tianjin Medical University, Tianjin, 300070 China

## Abstract

Magnolin is a multi-bioactive natural compound that possesses underlying anti-cancer properties. However, the mechanisms underlying remain to be elucidated. Here, we report the role of magnolin in suppressing human colorectal cancer (CRC) cells via activating autophagy and cell cycle arrest in vitro and in vivo. Pre-treatment of cells with specific autophagy inhibitor (3-methyladenine) or knockdown of endogenous LC-3B by siRNA significantly abrogates magnolin-induced cell cycle arrest. Molecular validation mechanistically shows that magnolin-induced autophagy and cell cycle arrest in CRC cells is correlated with decreased transcriptional levels of leukemia inhibitory factor (LIF), and we further find that inhibition of LIF decreases phosphorylation level of Stat3 and represses transcriptional expression of Mcl-1. Furthermore, magnolin-induced autophagy and cell cycle arrest suppress the growth of xenograft colorectal tumors without apparent toxicity. Finally, we evaluate the clinical correlation of LIF/Stat3/Mcl-1 in CRC patient tissues. As expected, LIF, p-Stat3, and Mcl-1 levels are high in CRC tissue but are scarcely found in normal colon tissue. High positive expressions of LIF or Mcl-1 are associated with poor prognosis. Doubly positive cases have shown the worst outcome. Taken together, our results have clarified a novel molecular mechanism whereby magnolin induces autophagy and cell cycle arrest through LIF/Stat3/Mcl-1 pathway in CRCs. Our results also have revealed that magnolin has a promising therapeutic potential in CRCs.

## Introduction

Colorectal cancer (CRC) is one of the most commonly diagnosed cancers and leading causes of cancer-related mortality worldwide^1,2^. Despite the benefits of early screening, surgery and other localized therapeutic intervention, the current 5-year survival rate for advanced CRC patients is only 8%^3^. There is a severe lack of highly reliable strategies for better clinical prevention/therapy. Regorafenib, a novel oral multikinase spectrum inhibitor, has demonstrated effectiveness in patients with chemorefractory metastatic CRC, which progresses though every available standard therapy has been applied^4^. However, the use of regorafenib is clinically hampered by its modest efficacy in unselected patient populations, serious side-effects, and high drug costs^4,5^. Thus, in order to improve patient outcomes, the development of novel effective and promising strategies for advanced CRC treatment is still urgently needed.

Natural products with highly diverse bioactivities and functions play a dominant role in the discovery of lead compounds for cancer treatment and prevention. Magnolin, an active furofuranoid lignans from *Magnolia biondii*, exhibits various biological activities, including anti-inflammatory activity, anti-cancer, anti-oxidative, and vasodilatory effects^6–10^. Although the targets or effectors of magnolin are not well-defined, the widespread biological activities and low toxic side-effects of magnolin render it a promising drug candidate in clinical development. Recent studies have demonstrated that magnolin markedly suppresses cell proliferation and transformation by targeting ERKs activities^7,11^. However, the defined molecular mechanisms of magnolin on tumorigenesis remain elusive.

In this study, we have demonstrated that magnolin suppresses the growth of CRC by inducing autophagy and cell cycle arrest in vitro and in vivo. Molecular validation mechanistically demonstrates that magnolin-induced autophagy and cell cycle arrest in CRC cells is associated with decreased transcriptional levels of leukemia inhibitory factor (LIF), and we further find that inhibition of LIF decreases phosphorylation level of Stat3 and represses transcriptional expression of Mcl-1. Furthermore, magnolin-induced autophagy and cell cycle arrest suppress the growth of xenograft colorectal tumors without remarkable toxicity. Finally, we evaluate the clinical correlation of LIF/Stat3/Mcl-1 in CRC patient tissues. As expected, LIF, p-Stat3, and Mcl-1 levels are high in CRC tissue but are scarcely found in normal colon tissue. High positive expressions of LIF or Mcl-1 are associated with poor prognosis. Doubly positive cases have shown the worst outcome. Taken all together, these results suggest that magnolin serves as a novel and promising drug candidate via blocking LIF/Stat3/Mcl-1 axis for future CRC therapy.

## Results

### Magnolin inhibits growth and induces cell cycle arrest in CRC cells

**Fig. 1:**
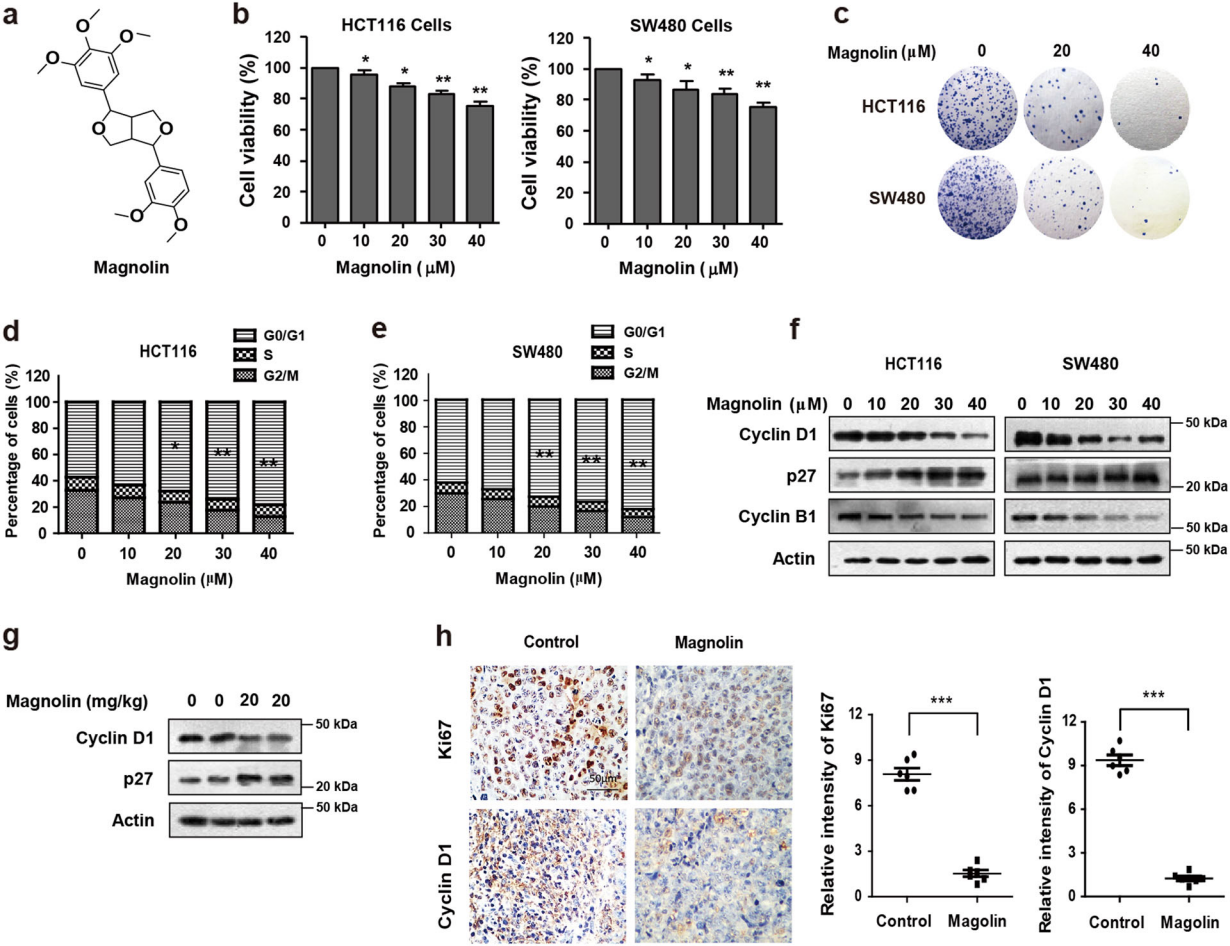
Magnolin inhibits growth and induces cell cycle arrest in CRC cells. **a** Chemical structure of magnolin. **b** Cell viability was examined using MTT assay in HCT116 and SW480 cells treated with magnolin at different concentrations for 48 h. **c** The clonogenicity of HCT116 and SW480 cells were detected after treatment with magnolin at different concentrations for 14 days. **d**, **e** HCT116 and SW480 cells were treated with indicated concentrations of magnolin for 48 h. Cell cycle distribution was determined by flow cytometer. **f** The protein levels of Cyclin D1, p27, and Cyclin B1 were determined by western blot assays. **g** Cyclin D1 and p27 levels in xenograft tumors were examined by western blot assays. **h** Ki67 and Cyclin D1 expressions in xenograft tumors were examined by IHC staining. Representative images were conducted as indicated. ***P < 0.001; Scale bars, 50 μm. For (**b**), (**d**) and (**e**), data are shown as mean ± s.d. (*n* = 3); *P < 0.05; **P < 0.01 compared with control (Student’s *t* test). For (**g**) and (**h**), data are shown as mean ± s.d. (*n* = 9). All the western data shown are representative of at least three independent experiments

The chemical structure of magnolin is shown in Fig. 1a. The MTT assay was used to examine the cytotoxic effects of magnolin against two typical CRC cell lines (HCT116 and SW480). As shown in Fig. 1b, cell viability of CRC cell lines was remarkably decreased dose-dependently by magnolin (0–40 μM) for 48 h. Consistently, as evidenced by reduced clonogenicity (Fig. 1c), magnolin significantly inhibited cell proliferation in CRC cells. To explore cell apoptosis induction effect of magnolin on CRC cells, we performed employing Annexin V staining and western blot assays. Magnolin slightly promoted CRC cells apoptosis (Supplementary Fig. 1a,b). To explore cell cycle arrest induction effect of magnolin on CRC cells, we performed flow cytometry detection. As shown in Fig. 1d, e, magnolin markedly increased cell number at G0/G1 phase after 48 h exposure, accompanied by reduced cell number at G2/M phase in HCT116 and SW480 cells. The cell cycle arrest effects were further confirmed by employing western blot assays. Along with activation of p27, Cyclin D1 and Cyclin B1 were markedly decreased by magnolin dose-dependently (Fig. 1f).

In order to further investigate the effect of magnolin on CRC cell cycle arrest in vivo, western blot assays and IHC staining detection were performed in CRC xenograft tumors. As shown in Fig. 1g, magnolin treatment significantly decreased Cyclin D1 levels and clearly increased p27 levels in HCT116 tumors compared with that of the control group. Consistently, a similar trend was observed in Cyclin D1 and p27 staining (Fig. 1h and Supplementary Fig. 1c). Furthermore, we detected xenografts by Ki67 staining to assess the change of tumor proliferation status. There was a marked decrease in the percentage of Ki67-positive staining in magnolin-treated tumors, as compared with that of control tumors (Fig. 1h). Collectively, these results suggest that magnolin inhibits the proliferation and induces cell cycle arrest of CRC in vitro and in vivo.

### Magnolin promotes autophagy in CRC cells

**Fig. 2:**
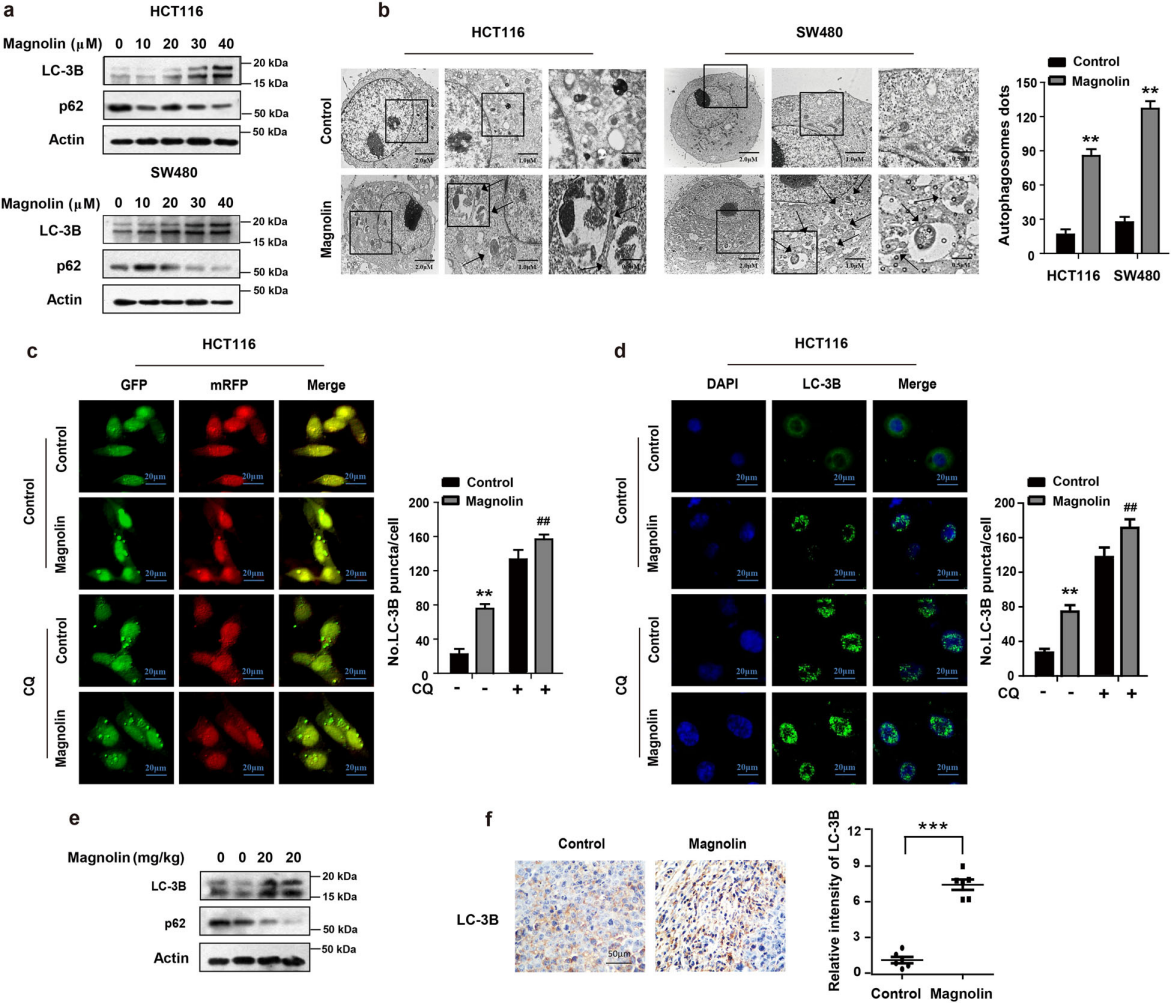
Magnolin promotes autophagy in CRC cells. **a** Western blotting analysis shows that the protein expression of LC-3B and p62 was measured in HCT116 and SW480 cells treated with indicated concentrations of magnolin for 48 h. **b** Autophagy was determined after treatment with DMSO or magnolin (40 μM) for 48 h by transmission electron microscopy. Right, quantitative analysis of autophagosomes. Data are shown as mean ± s.d. (*n* = 3); **P < 0.01 compared with control. **c**, **d** Cells were treated with magnolin with or without CQ respectively. **c** Cells were transfected with a reporter plasmid (mRFP-GFP-LC3), followed by a confocal laser scanning microscope. Right, total number of endogenous LC3 puncta per cell. **d** The endogenous LC-3B puncta formation was measured by IF analysis. Right, quantitative analysis of autophagosomes. For (**c**) and (**d**), data are shown as mean ± s.d. (*n* = 3); **P < 0.01 compared with control; ^##^*P* < 0.01 compared with cells treated with magnolin (Student’s *t* test). Scale bar, 20 μm. **e** Xenograft tumors were examined at the levels of LC-3B and p62 by western blot assays. **f** LC-3B expression in xenograft tumors was determined by IHC staining. Representative images were conducted as indicated. ***P < 0.001; Scale bar, 50 μm. For (**e**) and (**f**), data are shown as mean ± s.d. (*n* = 9). All the western data shown are representative of at least three independent experiments

Autophagy, one mode of programmed cell death, plays a critical role in cancer development and progression^12,13^. In this present study, we first determined the protein levels of two specific autophagy markers, LC-3B and p62, by western blot assays. As shown in Fig. 2a and Supplementary Fig. 2a and b, the result demonstrated that magnolin significantly increased the expression of LC-3B-II, while markedly decreased the expression of p62 in a dose-dependent manner. To further verify magnolin-induced autophagy, we analyzed the cellular ultrastructure of CRC cells by transmission electronic microscopy. As shown in Fig. 2b, there was a dramatic accumulation of double membrane vesicles containing subcellular materials in magnolin-treated cells. Using a tandem mRFP-GFP-tagged LC-3, we found strong green fluorescent (GFP-tagged LC-3), with red dots (indicating autolysosomes) and yellow dots (indicating autophagosomes) being generated. Combinatorial treatment with the specific lysosomal inhibitor chloroquine (CQ) and magnolin exposure resulted in a further increased conversion of LC3-I to LC-3-II, promotion of LC-3 puncta and lipidation, and accumulation of autophagosomes (Fig. 2c, d and Supplementary 2c,d). Moreover, western blot assays showed that CQ promoted LC-3B conversion, abrogated p27 induction and suppressed p62 and Cyclin D1 downregulation in magnolin-treated CRC cells (Supplementary 2e,f). Furthermore, we investigated the effect of magnolin on CRC autophagy in vivo by western blot assays and IHC staining. As shown in Fig. 2e, magnolin treatment strongly increased the expression level of LC-3B-II and dramatically decreased the expression level of p62 in HCT116 tumors compared to that of the control group by western blot assays. Consistently, IHC staining demonstrated that positive staining of LC-3B was much stronger, but positive staining of p62 and NBR1 were much weaker in magnolin-treated xenografts tumors compared with control xenografts tumors. (Fig. 2f and Supplementary Fig. 2g). Collectively, these results suggest that magnolin promotes autophagy in CRC cells.

### Inhibition of autophagy blocks magnolin-induced cell cycle arrest

**Fig. 3:**
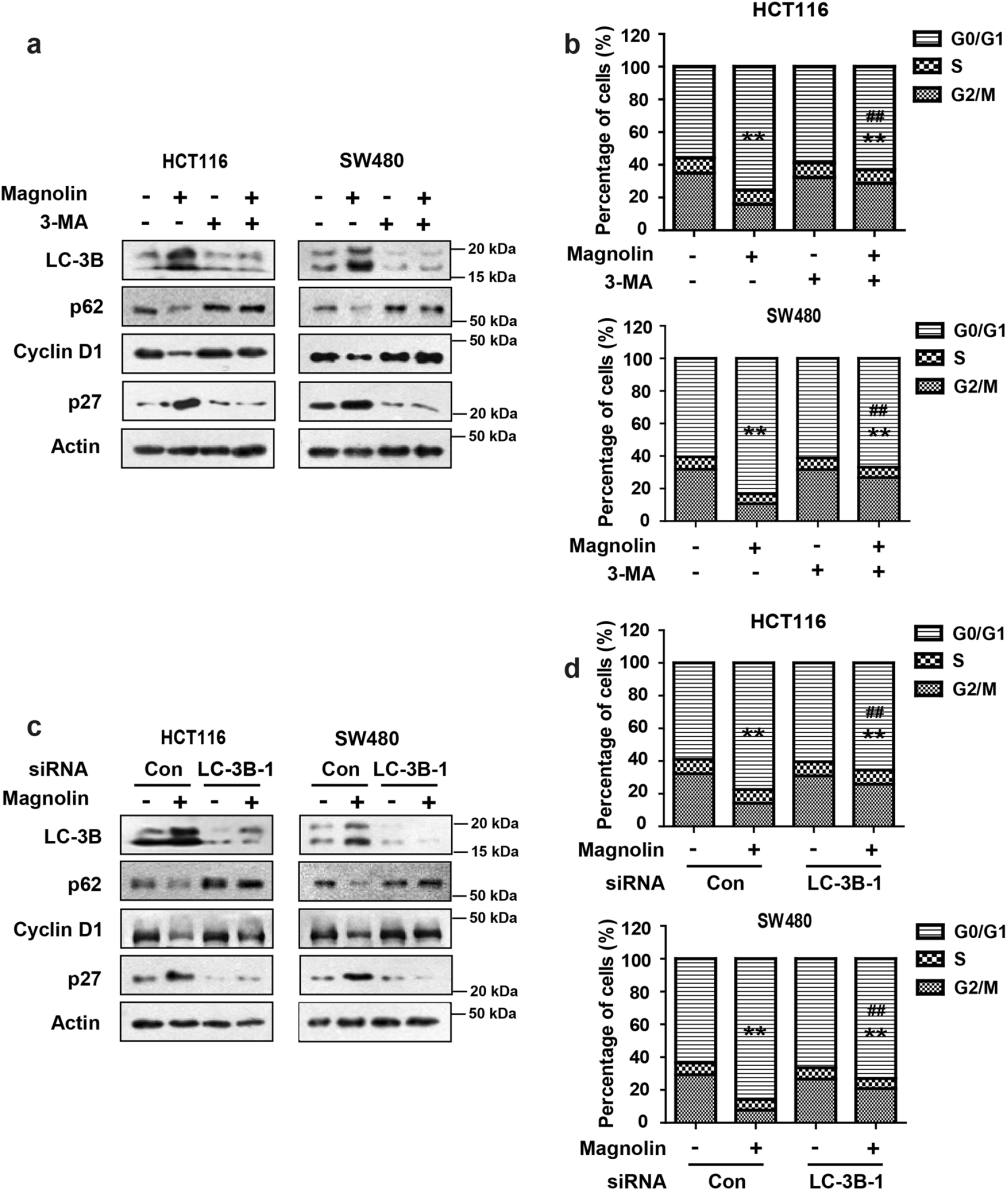
Inhibition of autophagy blocks magnolin-induced cell cycle arrest. **a**, **b** Cells were treated with magnolin with or without 3-MA respectively. **a** The levels of LC-3B, p62, Cyclin D1, and p27 proteins were examined by western blot assays. **b** The cell cycle distribution was determined by flow cytometer. **c**, **d** Cells were transfected with control siRNA or siRNA against LC-3B followed by magnolin treatment. **c** The levels of LC-3B, p62, Cyclin D1, and p27 proteins were examined by western blot assays. **d** The cell cycle distribution was determined by flow cytometer. For (**b**) and (**d**), data are shown as mean ± s.d. (*n* = 3); **P < 0.01 compared with control; ^##^*P* < 0.01 compared with control or si.control transfected cells treated with magnolin (Student’s *t* test). All the western data shown are representative of at least three independent experiments

Autophagy plays a pivotal role in maintaining cell growth and survival by regulating cell cycle progression^14,15^. To determine the relationship between autophagy and cell cycle arrest induced by magnolin on CRC cells, HCT116 and SW480 cells were treated with magnolin for 48 h along with or without the specific autophagy inhibitor 3-Methyladenine (3-MA). LC-3B, p62, p27, and Cyclin D1 protein levels were analyzed by western blot assays. As shown in Fig. 3a, in magnolin-treated CRC cells, 3-MA abrogated LC-3B conversion and p27 induction and suppressed p62 and Cyclin D1 downregulation. Furthermore, the cell cycle arrest effect of magnolin was also clearly blocked when combined with 3-MA (Fig. 3b). Consistently, knockdown of endogenous LC-3B by siRNA in CRC cells dramatically suppressed magnolin-induced autophagy and cell cycle arrest in CRC cells (Fig. 3c, d and Supplementary Fig. 3a,b). Furthermore, knockdown of endogenous Atg3 in CRC cells dramatically abrogated magnolin-inhibited the proliferation (Supplementary Fig. 3c−e). Taken together, these results show that inhibition of autophagy could block magnolin-regulated cell cycle arrest.

### Magnolin inhibits Mcl-1 through inactivation of the LIF signaling

**Fig. 4:**
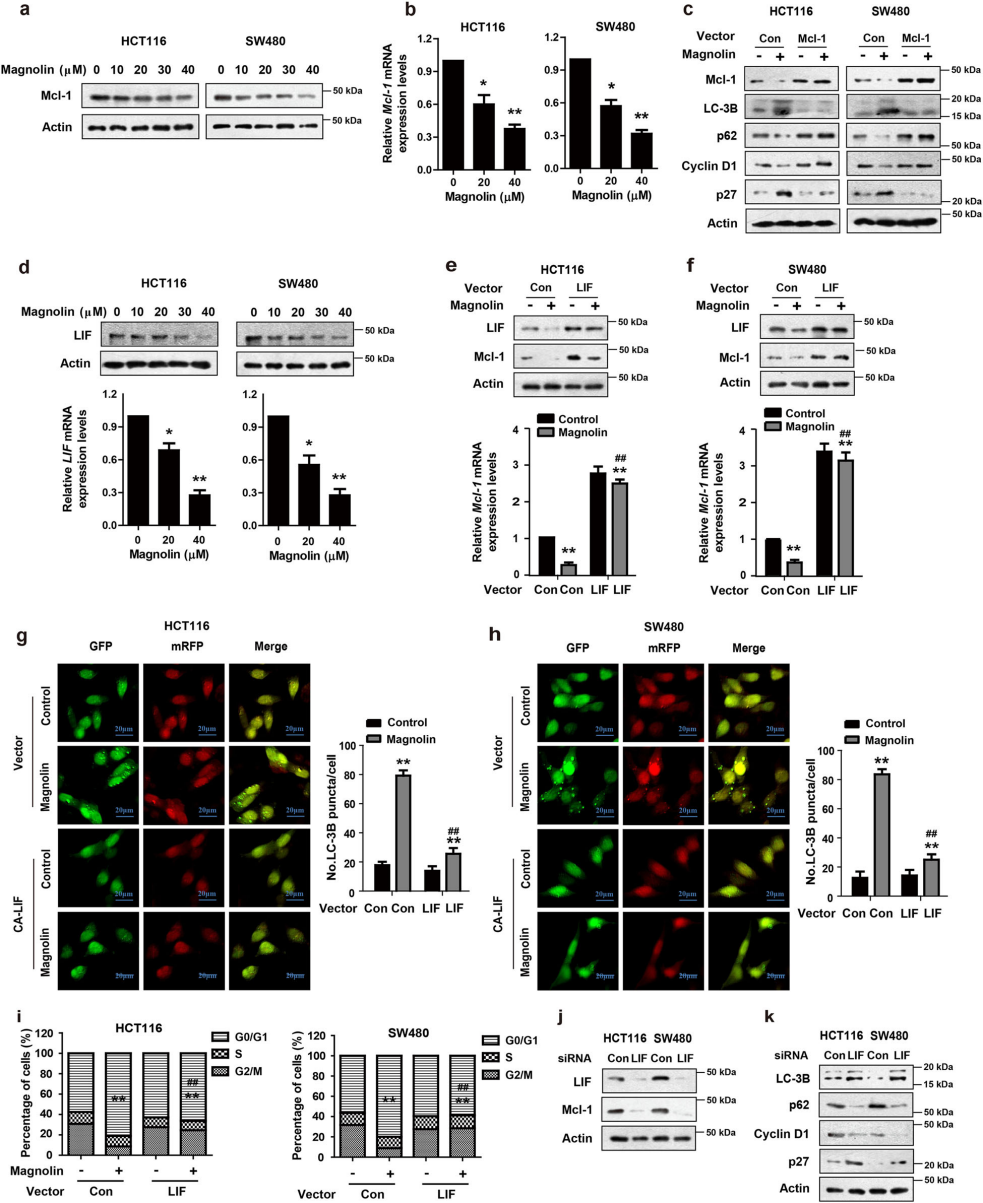
Magnolin inhibits Mcl-1 through inactivation of the LIF signaling. **a, b** HCT116 and SW480 cells were treated with indicated concentrations of magnolin for 48 h. **a** The protein levels of Mcl-1 were determined by western blot assays. **b** The mRNA levels of *Mcl-1* were detected by real-time PCR. **c** Cells were transfected with Mcl-1 (Mcl-1 Vec) or empty vector (Control Vec) and followed by magnolin treatment. The levels of Mcl-1, LC-3B, p62, Cyclin D1, and p27 proteins were detected by western blot assays. **d** The protein and mRNA levels of LIF were detected by western blot assays and real-time PCR. **e**–**i** Cells were transfected with LIF (LIF Vec) or empty vector (Control Vec) and followed by magnolin treatment. **e**, **f** The protein levels of LIF and Mcl-1 were determined by western blot assays. The mRNA levels of *Mcl-1* were detected by real-time PCR. **g**, **h** Cells were transfected with a reporter plasmid (mRFP-GFP-LC3), followed by a confocal laser scanning microscope. Scale bar, 20 μm. **i** The cell cycle distribution was determined by flow cytometer. **j**, **k** Cells were transfected with control siRNA or siRNA against LIF. **j** The levels of LIF and Mcl-1 proteins were determined by western blot assays. **k** The levels of LC-3B, p62, Cyclin D1, and p27 proteins were detected by western blot assays. For (**b**) and (**d**), data are shown as mean ± s.d. (*n* = 3); *P < 0.05; **P < 0.01 compared with control (Student’s *t* test). For (**e**–**i**), data are shown as mean ± s.d. (*n* = 3); **P < 0.01 compared with vector control transfected cells; ^##^*P* < 0.01 compared with vector control transfected cells treated with magnolin (Student’s *t* test). All the western data shown are representative of at least three independent experiments

It has been reported that Mcl-1 plays key roles in the regulation of cell life and death^16,17^. In this study, we found that magnolin significantly downregulated the expression of Mcl-1 at both mRNA and protein levels (Fig. 4a, b). Ectopic Mcl-1 expression abolished LC-3B conversion and p27 induction and prevented p62 and Cyclin D1 downregulation in magnolin-treated CRC cells (Fig. 4c and Supplementary Fig. 4a,b). Furthermore, Mcl-1 overexpression suppressed magnolin-regulated autophagic flux (Supplementary Fig. 4c,d) and cell cycle arrest (Supplementary Fig. 4e,f) in CRC cells. LIF is an important regulator and is frequently overexpressed in different human tumor types. In the present study, we found that LIF mRNA and protein levels were markedly decreased in response to magnolin dose-dependently (Fig. 4d). Ectopic LIF expression clearly increased Mcl-1 mRNA and protein levels in magnolin-treated CRC cells (Fig. 4e, f). Moreover, LIF overexpression also suppressed magnolin-induced autophagic flux (Fig. 4g, h) and cell cycle arrest (Fig. 4i) in CRC cells. Consistently, knockdown of endogenous LIF by siRNA markedly decreased Mcl-1 mRNA and protein levels (Fig. 4j and Supplementary Fig. 5a), and knockdown of endogenous LIF clearly increased conversion of LC-3B and p27 induction and promoted p62 and Cyclin D1 downregulation (Fig. 4k and Supplementary Fig. 5b). Collectively, these results demonstrate that magnolin inactivates the LIF signaling pathway, which in turn downregulates Mcl-1 and induces autophagy and cell cycle arrest of CRC.

### LIF promotes Stat3 phosphorylation to block magnolin-induced autophagy and cell cycle arrest

**Fig. 5:**
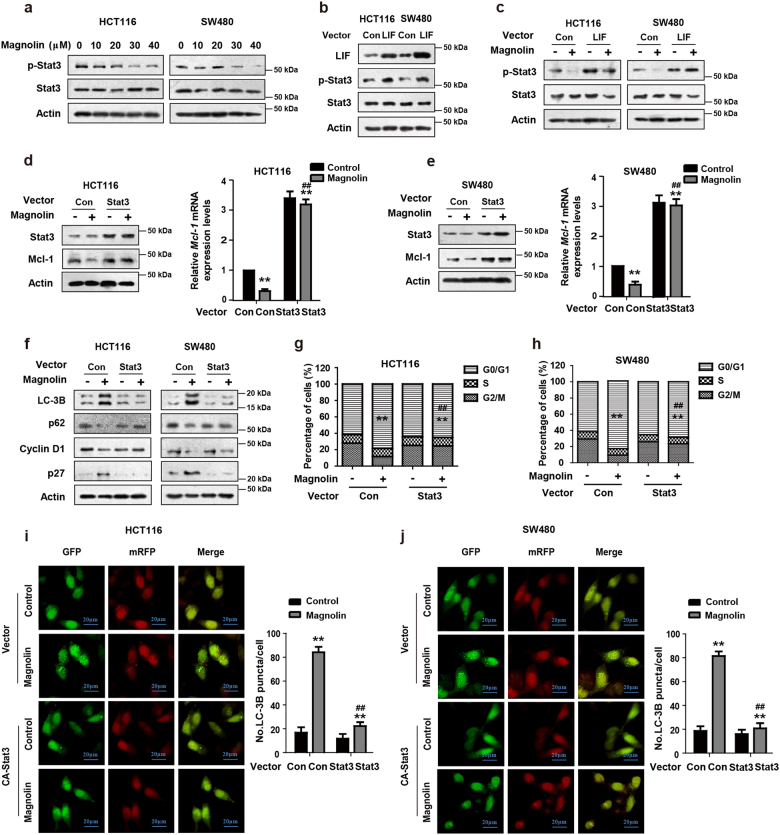
LIF promotes Stat3 phosphorylation to block magnolin-induced autophagy and cell cycle arrest. **a** HCT116 and SW480 cells were treated with indicated concentrations of magnolin for 48 h. The protein levels of p-Stat3 were determined by western blot assays. Total Stat3 expressions were detected as the internal control. **b** Cells were transfected with LIF (LIF Vec) or empty vector (Control Vec). The levels of LIF, p-Stat3, and Total Stat3 proteins were detected by western blot assays. **c** Cells were transfected with LIF (LIF Vec) or empty vector (Control Vec) and followed by magnolin treatment. The levels of p-Stat3 and Total Stat3 proteins were determined by western blot assays. **d**–**j** Cells were transfected with Stat3 (LIF Vec) or empty vector (Control Vec) and followed by magnolin treatment. **d**, **e** The protein levels of Stat3 and Mcl-1 were determined by western blot assays. The mRNA levels of *Mcl-1*were detected by real-time PCR. **f** The protein levels of LC-3B, p62, Cyclin D1, and p27 were detected by western blot assays. **g**, **h** The cell cycle distribution was determined by flow cytometer. **i**, **j** Cells were transfected with a reporter plasmid (mRFP-GFP-LC3), followed by a confocal laser scanning microscope. For (**d**, **e**) and (**g**–**j**), data are shown as mean ± s.d. (*n* = 3); **P < 0.01 compared with vector control transfected cells; ^##^*P* < 0.01 compared with vector control transfected cells treated with magnolin (Student’s *t* test). All the western data shown are representative of at least three independent experiments

Stat3 is a transcription factor that regulates downstream target genes and plays a major role in tumor survival and oncogenesis^18,19^. Interestingly, we found that magnolin dramatically inhibited the phosphorylation level of Stat3, but there was no obvious change in its total expression in CRC cells (Fig. 5a). The inhibition of p-Stat3 by magnolin was mediated through LIF, and ectopic LIF expression induced Stat3 phosphorylation (Fig. 5b, c). To investigate whether Stat3 mediates magnolin-induced autophagy and cell cycle arrest in CRC cells, the protein and mRNA levels of Mcl-1 were detected by western blot assays and real-time PCR, respectively. As shown in Fig. 5d, e, ectopic Stat3 expression clearly blocked magnolin-inhibited Mcl-1 at both mRNA and protein levels in CRC cells. Furthermore, Stat3 overexpression strongly blocked magnolin-induced autophagy and cell cycle arrest (Fig. 5f−j). Collectively, these data suggest that LIF promotes Stat3 phosphorylation to block magnolin-induced autophagy and cell cycle arrest.

### Magnolin inhibits growth and development in colorectal HCT116 xenograft tumors

**Fig. 6:**
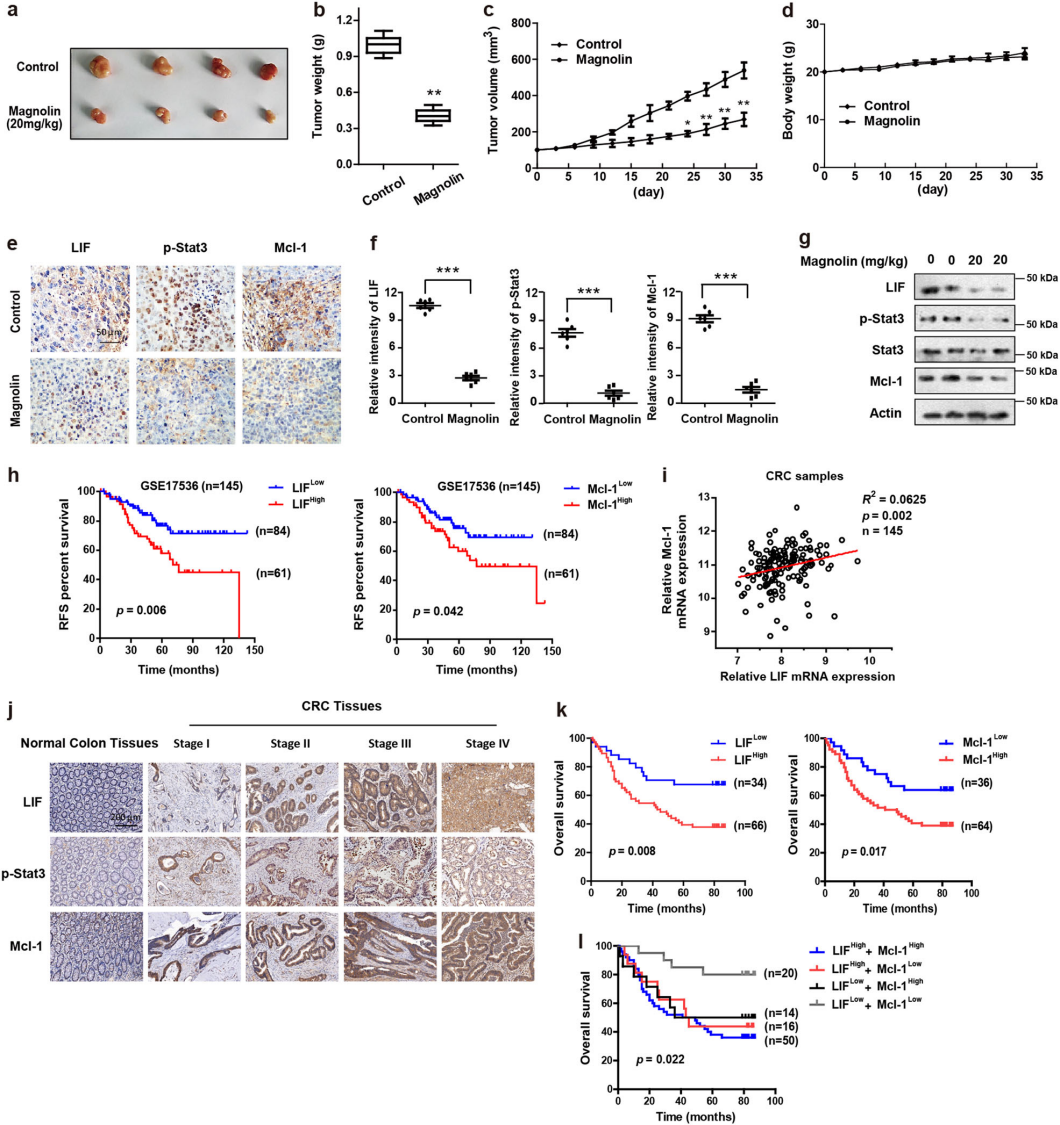
Magnolin inhibited growth and development in colorectal HCT116 xenograft tumors. **a**–**g** BALB/c nude mice were inoculated with HCT116 cells and treated with magnolin or vehicle. **a** Tumors were isolated and photographed. **b** Tumors were weighted. **c** Tumor volumes were measured every 3 days. **d** Bodies were weighted. **e**, **f** LIF, p-Stat3, and Mcl-1 expressions were determined by IHC staining in xenograft tumors. Representative images were conducted as indicated. ***P < 0.001; Scale bars, 50 μm. **g** LIF, p-Stat3, Stat3, and Mcl-1 levels were determined in xenograft tumors by western blot assays. Western data shown are representative of at least three independent experiments. **h** Kaplan–Meier plots of the relapse-free survival (RFS) of CRC patients, stratified by expression of LIF or Mcl-1. Data obtained from the publically available datasets (GSE17536). **i** The relevance between LIF and relative expression of Mcl-1 in clinical CRC samples. **j** Representative micrographs of LIF, p-Stat3, and Mcl-1 expression in CRC and normal colon tissues, as analyzed by IHC. Scale bars, 200 μm. **k** The overall survival of CRC patients with LIF and Mcl-1 expression, as well as combined expression of LIF and Mcl-1 (**l**) are analyzed by the Kaplan–Meier estimates and the log-rank test. For (**a**–**g**), data are shown as mean ± s.d. (*n* = 9). *P < 0.05; **P < 0.01 compared with control (Student’s *t* test)

To investigate whether magnolin inhibits CRC growth and development in vivo, we established a colon tumor xenograft model by injecting human HCT116 cells subcutaneously into nude mice. As shown in Fig. 6a, macroscopically, the size of magnolin-treated tumors was markedly reduced compared with that of the control group. Consistently, tumor weight in magnolin-treated mice was much smaller than that of the control group (Fig. 6b). Xenografts treated with magnolin continued to grow but at a considerably slower rate than those treated with vehicle (Fig. 6c). However, there was no noticeable difference in body weight (Fig. 6d) between the control and magnolin-treated groups. IHC staining demonstrated that positive staining of LIF, p-Stat3, and Mcl-1 was much lower in magnolin-treated xenografts tumors compared with control xenografts tumors (Fig. 6e, f). Consistently, magnolin treatment inhibited Stat3, and attenuated protein levels of LIF and Mcl-1 in HCT116 xenograft tumors (Fig. 6g).

### Coexpression of LIF, p-Stat3, and Mcl-1 correlates with a poor prognosis in CRC patients

To further evaluate the clinical correlation of LIF/Stat3/Mcl-1 in CRC patient tissues, we subjected them to Kaplan–Meier survival analysis in the publically available dataset of CRC patients (GSE17536). Data showed that patients with higher expressions of LIF or Mcl-1 displayed poorer relapse-free survival rate (Fig. 6h). Spearman correlation analysis demonstrated that the expression of LIF in CRC tissues was positively associated with the expression of Mcl-1 (Fig. 6i). Subsequently, we determined primary tumor samples from 100 CRC patients by IHC. LIF, p-Stat3, and Mcl-1 levels were high in CRC tissue but were scarcely found in normal colon tissue (Fig. 6j). High positive expressions of LIF or Mcl-1 proteins were associated with poor prognosis (Fig. 6k). Doubly positive cases showed the worst outcome (Fig. 6l). Collectively, these results suggest that LIF/Stat3/Mcl-1 axis could be prognosis markers for poor survival in CRC patients.

Taken together, our results suggest that the LIF/Stat3/Mcl-1 axis plays a key role in magnolin-induced autophagy and cell cycle arrest in CRC.

## Discussion

Magnolin, an active furofuranoid lignans compound, has been reported to possess broad biological activities. Recent studies have demonstrated that magnolin shows remarkable efficacy in inhibiting tumor growth^7,8,11,20^. However, the exact molecular mechanisms have not been elucidated. In this study, we have demonstrated that magnolin represses Stat3/Mcl-1 signaling via targeting LIF, and thereby inducing autophagy and cell cycle arrest, leading to the suppression of the growth of CRCs in vitro and in vivo.

Cell cycle progression is monitored strictly and checkpoints during phase transitions of the cell cycle ensure healthy cell progression and proliferation^21,22^. Loss of cell cycle control is one of the typical characteristics of tumorigenesis. Progression through the cell cycle is known to be controlled by cyclins, cyclin-dependent kinase (CDKs) and cyclin-dependent kinase inhibitors (CKIs), which play essential roles in the G1/S phase transition. And the Cyclin D1 and p27 proteins specifically serve as key regulators of early cell fate to coordinate entry into S phase^23–25^. We have found that magnolin induces G1 phase arrest accompanied by decreased proportion of G2 phase in CRC cells. We have also demonstrated that magnolin treatment markedly upregulates the protein expression level of p27, while decreases the protein expression level of Cyclin D1. Cyclin B1, as a tumor antigen, is a key mitotic cyclin in the G2/M phase transition of the cell cycle^24,25^. In this study, we have found that magnolin treatment significantly decreases the protein expression level of Cyclin B1. Together, these results suggest that magnolin induces cell cycle arrest in CRC cells.

Autophagy is evolutionarily conserved, and it participates in the fusion between double-membraned autophagosomes and lysosomes to form autolysosomes^26–28^. Transmission electron microscopy (TEM) is considered as one of the most convincing and sensitive methods to be used to examine whether autophagic compartments are formed, which subsequently engulf cytoplasmic components and organelles, including endoplasmic reticulum, ribosomes and mitochondria^29^. In this study, we have found that magnolin accumulates the formation of autophagic vacuoles (AVs) by TEM. A lipidated form of LC-3B, LC-3B-II which is localized on the AVs, has been used as a typical marker of autophagosomes. In this study, we have found that magnolin markedly promotes LC-3 lipidation and the formation of positive autophagic puncta, increases LC-3B-II/LC-3B-I ratio, and induces autophagic flux formation. P62 represents an autophagy adaptor, being capable of directly binding ubiquitin and autophagy components. P62 expression is generally inversely proportional to autophagic degradation, and meanwhile serves as a good indicator of autophagy flux. We have found that magnolin significantly inhibits the protein expression level of p62. Altogether, these results markedly suggest that magnolin induces autophagy in CRC cells.

Recent numerous lines of evidence suggest that manipulation of autophagy may provide important insights into the prevention of cancer development and progression as well as the improvement of cancer therapy^13,30^. Autophagy deficiency, which is associated with activated DNA damage response and genomic instability, is usually more likely to result in tumor formation and progression^31,32^. Paradoxically, however, autophagy can also conversely promote the survival and proliferation of cancer cells under environmental and intracellular stress conditions, such as inadequate nutrient supply, hypoxia, chemotherapy and radiotherapy, thus accelerating tumor growth^33,34^. Therefore, autophagy has a dual role, as it can either promote or suppress cancer for therapeutic purposes depending on the circumstances.

Autophagy has been found to exhibit essential roles in maintaining cell survival via cell cycle-regulating. Previous studies have demonstrated that autophagy induces cell cycle arrest in human glioblastoma cells through inhibiting cancer cell growth^35^, and some researchers have found that induction of autophagy by boswellic acid analog may promote G2/M arrest and inhibit tumor growth in human pancreatic cancer cells^36^. Furthermore, overexpression of cell cycle inhibitors (p27, CDK-1) is sufficient to promote autophagy^37^. It seems necessary to further clarify the complexity of the interplay between cell cycle and autophagy under stress conditions. In this study, we have found that pre-treatment of cells with 3-MA or knockdown of endogenous LC-3B by siRNA markedly blocks magnolin-induced cell cycle arrest. Therefore, magnolin may induce cell cycle arrest via triggering autophagy, which results in suppressing the growth of CRC in vitro and in vivo.

Mcl-1, a member of Bcl-2 family proteins, has been found frequently overexpressed in multiple cancer types. Recent studies suggest that Mcl-1 can inhibit autophagy and play critical roles in cancer cell survival and death^17,38^. In this present study, we have demonstrated that magnolin transcriptionally suppresses Mcl-1 expression in CRC cells. Consistently, ectopic Mcl-1 expression clearly inhibits magnolin-induced autophagy and cell cycle arrest in CRC cells. Stat3, a member of the STAT family of transcription factors, mediates cell growth, differentiation and survival signals in many types of cells^18,19,39^. Previous studies have shown that IL-6-induced Stat3 signaling upregulates Mcl-1 transcription in cholangiocarcinoma cells. In this study, we have found that magnolin dramatically decreases phosphorylation level of Stat3. Ectopic Stat3 expression upregulates Mcl-1 transcription and strongly blocks magnolin-induced autophagy and cell cycle arrest in CRC cells. Collectively, these results suggest that Stat3/Mcl-1 pathways play a dominant role in magnolin-induced autophagy and cell cycle arrest.

LIF, a member of the Interleukin-6 family, is a multifunctional cytokine that exerts a variety of effects on cell and tissue types. Recent studies have demonstrated that LIF promotes tumor development and progression^40,41^. Overexpression of LIF significantly enhances proliferation, growth, and metastasis of both cultured human cancer cells and xenografts^41–43^. Moreover, overexpression of LIF has been observed most frequently in many types of cancers, such as colorectal, lung, breast, melanoma and nasopharyngeal, head and neck cancer^41–45^. Our previous studies have found that LIF promotes p53-regulated CRC chemoresistance, and patients with higher LIF levels often have a poor prognosis^42^. Thus, targeting LIF has become a potential strategy in cancer therapy. In this present study, we have demonstrated that magnolin dramatically decreases the levels of LIF protein, and LIF expression markedly suppresses magnolin-induced autophagy and cell cycle arrest in CRC cells. Recent studies have demonstrated that LIF can selectively activate several signaling pathways, including JAK/Stat3, PI3K/Akt, MAPK, and mTOR, depending on cell type and tissue-specific manner^41,42^. In our previous studies, we have found that LIF negatively regulates p53 protein levels and function through Stat3/ID1/MDM2 axis activation in CRCs^42^. Results from this study have clearly shown that ectopic LIF expression in CRC cells significantly blocks the inhibitory effect of magnolin on Stat3/Mcl-1 pathway. Clinical studies have shown that LIF expression is positively associated with the Mcl-1, and high positive expressions of LIF or Mcl-1 are associated with poor prognosis. Collectively, our results suggest that the LIF/Stat3/Mcl-1 axis plays a key role in magnolin-regulated autophagy and cell cycle arrest in CRCs.

In summary, these results elucidate that magnolin promotes autophagy and cell cycle arrest through LIF/Stat3/Mcl-1 pathway, which in turn prevents the tumor growth of CRC. These findings indicate that magnolin might represent a promising candidate drug for future CRC therapeutics.

## Materials and methods

### Cell culture and cell treatments

Human CRC cell lines HCT116 and SW480 were obtained from ATCC in April 2016. The cells being used were used within 1 month after resuscitation. The cell lines were identified using a short tandem repeat analysis. Mycoplasma contamination was excluded in these cell lines. Cells were maintained at 37 °C in RPMI-1640 supplemented with 10% FBS and 1% penicillin/streptomycin in a humidified incubator under 5% CO_2_. Expression vectors of human LIF, Stat3, and Mcl-1 were designed and purchased from Servicebio Technologies (Wuhan, China). For siRNA knockdown, siRNA oligos against LC-3B, Atg3, and LIF were obtained from Hanbio Biotechnology (Shanghai, China). 3-MA and chloroquine (CQ) were obtained from Selleck (London, ON, Canada). Magnolin with greater than 98% purity was obtained from Shanghai Yuanye Bio-Technology (Shanghai, China). 3-(4, 5-dimethyl-2-thiazolyl)-2, 5-diphenyl-2-H-tetrazolium bromide (MTT) and 4, 6-diamidino-2-phenylindole (DAPI) reagents were obtained from Sigma-Aldrich (St. Louis, MO, USA).

### Cell viability assay

Cell viability was measured using an MTT assay. In brief, human CRC cells (5×10^3^ cells/well) were treated with magnolin at different concentrations for 48 h, and further incubated with MTT solution at 37 °C for 4 h. Then, medium was removed. DMSO (100 μl) was added and acquired by a microplate reader at 570 nm. For blocking study, cells were pre-cultured with 1 mM 3-MA for 1 h, and then treated with 40 μM magnolin for 48 h.

### Cell cycle distribution analysis

Cell cycle distribution was determined by flow cytometer as previously described^46^. Briefly, human CRC cells were maintained in six-well plates with different concentrations of magnolin for 48 h. The cells were harvested and fixed with 75% ethanol, and re-suspended in 50 μg/ml of PI staining buffer for 15 min at 37 °C. Cell cycle distribution was analyzed by flow cytometer FACS Verse (BD Biosciences, San Jose, CA, USA).

### Quantitative real-time PCR

Total RNA was purified as previously described^47^. Real-time PCR was done in triplicate with TaqMan or SYBGreen PCR mixture (Life technology, Foster City, CA, USA). The LIF probe was purchased from Life technology. The *Mcl-1* and *Actin* primers were synthesized from Sangon Biotech as follows: For *Mcl-1*, 5′-GGACATCAAAAACGAAGACG-3′ and 5′-GCAGCTTTCTTGGTTTATGG-3′; For *Actin*, 5′-GGACTTCGAGCAAGAGATGG-3′ and 5′-AGCACTGTGTTGGCGTACAG-3′.

### Colony formation assay

Colony formation assay was conducted as described above^48^. Human CRC cells were seeded into six-well plates and cultured overnight. Cells were then treated with different concentrations of magnolin. On day 14, colonies were fixed with 4% paraformaldehyde, and stained with 0.1% crystal violet. The colony number was counted in indicated time periods.

### Xenograft tumorigenicity assays

HCT116 cells (5×10^6^ in 0.2 ml PBS) were inoculated subcutaneously (via s.c. injection) into 7-week-old BALB/c female athymic nude mice (Taconic). When tumor volumes reached 100 mm^3^, mice were randomly assigned into two groups (*n* = 9, per group) and received vehicle or magnolin (20 mg/kg every other day) via i.p. injection for 33 days. Tumor volume and body weight were recorded every 3 days. The mouse experiments were performed according to protocols approved by the Animal Care and Use Committee of Tianjin University of Traditional Chinese Medicine. No specific exclusion or inclusion used for animal experiments.

### Immunofluorescence assay

Immunofluorescence (IF) analysis was conducted as described previously^48^. In brief, human CRC cells were fixed with 4% paraformaldehyde for 30 min, followed by incubation with 0.5% Triton X-100, and blocked with 5% BSA for 30 min at room temperature. The slides were incubated with anti-LC-3B antibody overnight at 4 °C, followed by incubation with Alexa-Fluor 488-conjugated goat anti-rabbit IgG antibody for 1 h at room temperature. Nuclear staining was then incubated with DAPI and visualized with an inverted fluorescent microscope (Carl Zeiss, Oberkochen, Germany).

### Tissue samples

The CRC tissue microarrays (*N* = 100) were obtained from Shanghai Outdo Biotech Company (Shanghai, China). These samples were collected from April 2008 to December 2008. All patients were followed up until July 2015. The studies were approved by the Ethics Committee of Taizhou Hospital of Zhejiang Province, and all patients provided written informed consent. The LIF, p-Stat3, and Mcl-1 staining results were classified according to the CRC cells staining intensity by four grades (0, negative; 1, weakly positive; 2, moderately positive; 3, strongly positive). We classified negative and weakly positive as low expressers, and moderately and strongly positive as high expressers.

### Database of colorectal cancer patients

Clinical data can be obtained via GEO with the publically available dataset (GSE17536)^49^. The expression level of LIF or Mcl-1 in CRC patients was analyzed by Kaplan−Meier estimate.

### Immunohistochemistry assay

Immunohistochemistry (IHC) analysis was conducted as reported previously^47^. The prepared sections were incubated with anti-Ki67, anti-Cyclin D1, anti-p27, anti-NBR1, anti-LC-3B, anti-LIF, anti-p-Stat3, and anti-Mcl-1 antibodies overnight at 4 °C, followed by adding biotin-conjugated secondary antibody. Images were visualized by a Leica DM4000B microscope (Leica, Wetzlar, Germany).

### Analysis of autophagic flux

To examine autophagic flux, cells were transfected with a reporter plasmid (mRFP-GFP-LC3) according to the instruction of the manufacturer (GeneChem, Shanghai, China). The transfected cells were treated with 40 μM of magnolin for 48 h. Cells then were fixed in 4% paraformaldehyde and washed in PBS. Finally, the GFP/mRFP images were obtained with a confocal laser scanning microscope (Olympus FV1000, Tokyo, Japan).

### Transmission electron microscopy

TEM was conducted as described previously^48^. In brief, human CRC cells were treated with 40 μM of magnolin for 48 h and fixed with 2% glutaraldehyde. The 50 nm ultrathin sections were cut with an ultramicrotome, contrasted with uranyl acetate/lead citrate, and determined with electron microscope Hitachi H-7650 (Hitachi, Tokyo, Japan).

### Western blot assays

Standard western blot assays were performed as described above^47,48^. Antibodies against SQSTM1/p62 (D5E2), LC-3B (D11), Cyclin B1 (Ser116), phospho-Stat3 (Tyr705), total Stat3, NBR1, and Mcl-1 were purchased from Cell Signaling Technology (Danvers, MA, USA). Antibodies against p27 and Cyclin D1 were purchased from BD Biosciences (San Jose, CA, USA). Anti-LIF (AF250-NA) antibody was purchased from R&D. Anti-β-actin (A5441) antibody was purchased from Sigma-Aldrich (St. Louis, MO, USA). Full scans of western blot assays are shown in Supplementary Fig. 6−11.

### Statistical analysis

The data were presented as mean ± s.d. The statistical significant differences were performed to analyze the results of animal experiments by the one- or two-way ANOVA and the unpaired Student’s *t* test. All other *P* values were evaluated using Student’s *t* test (unpaired, two tailed). Survival analysis was performed using the Kaplan−Meier estimates and the log-rank test. Experiments were performed in at least three independent experiments and the statistical variation (*P* < 0.05) was considered significant.

## Electronic supplementary material

Supplementary Data Cell Death and Disease

## References

[1] Siegel RL (2017). Colorectal cancer statistics. CA Cancer J. Clin..

[2] Arnold M (2017). Global patterns and trends in colorectal cancer incidence and mortality. Gut.

[3] Van Cutsem E (2016). ESMO consensus guidelines for the management of patients with metastatic colorectal cancer. Ann. Oncol..

[4] Khan K. et al. Functional imaging and circulating biomarkers of response to regorafenib in treatment-refractory metastatic colorectal cancer patients in a prospective phase II study. *Gut* (2017). 10.1136/gutjnl-2017-314178 10.1136/gutjnl-2017-314178 PMC6204951 28790159

[5] Sarfaty M. et al. Cost effectiveness of nivolumab in advanced renal cell carcinoma. *Eur. Urol*. **73**, 628–34 (2018). 10.1016/j.eururo.2017.07.041 28807351

[6] Kim JY (2009). In vitro anti-inflammatory activity of lignans isolated from Magnolia fargesii. Bioorg. Med. Chem. Lett..

[7] Lee CJ (2015). Magnolin inhibits cell migration and invasion by targeting the ERKs/RSK2 signaling pathway. Bmc Cancer.

[8] Huang Y (2017). Magnolin inhibits prostate cancer cell growth in vitro and in vivo. Biomed. Pharmacother..

[9] Wang F (2014). Magnolin protects against contrast-induced nephropathy in rats via antioxidation and antiapoptosis. Oxid. Med. Cell Longev..

[10] Ibarra-Alvarado C (2010). Vasoactive and antioxidant activities of plants used in Mexican traditional medicine for the treatment of cardiovascular diseases. Pharm. Biol..

[11] Lee CJ (2014). Targeting of magnolin on ERKs inhibits Ras/ERKs/RSK2-signaling-mediated neoplastic cell transformation. Carcinogenesis.

[12] Kimmelman AC, White E (2017). Autophagy and Tumor Metabolism. Cell Metab..

[13] Amaravadi R, Kimmelman AC, White E (2016). Recent insights into the function of autophagy in cancer. Genes Dev..

[14] Salazar-Roa M, Malumbres M (2017). Fueling the cell division cycle. Trends Cell Biol..

[15] An Z (2014). Autophagy is required for G1/G0 quiescence in response to nitrogen starvation in Saccharomyces cerevisiae. Autophagy.

[16] Perciavalle RM, Opferman JT (2013). Delving deeper: Mcl-1’s contributions to normal and cancer biology. Trends Cell Biol..

[17] Belmar J, Fesik SW (2015). Small molecule Mcl-1 inhibitors for the treatment of cancer. Pharmacol. Ther..

[18] Yu H, Lee H, Herrmann A, Buettner R, Jove R (2014). Revisiting STAT3 signalling in cancer: new and unexpected biological functions. Nat. Rev. Cancer.

[19] Lee HJ (2014). Drug resistance via feedback activation of Stat3 in oncogene-addicted cancer cells. Cancer Cell.

[20] Mukhija M, Lal Dhar K, Nath Kalia A (2014). Bioactive Lignans from Zanthoxylum alatum Roxb. stem bark with cytotoxic potential. J. Ethnopharmacol..

[21] Bertoli C, Skotheim JM, De Bruin RAM (2013). Control of cell cycle transcription during G1 and S phases. Nat. Rev. Mol. Cell Biol..

[22] Aarts M, Linardopoulos S, Turner NC (2013). Tumour selective targeting of cell cycle kinases for cancer treatment. Curr. Opin. Pharmacol..

[23] Sendinc E, Jambhekar A, Shi Y (2015). Remodeling your way out of cell cycle. Cell.

[24] Asghar U, Witkiewicz AK, Turner NC, Knudsen ES (2015). The history and future of targeting cyclin-dependent kinases in cancer therapy. Nat. Rev. Drug Discov..

[25] Diaz-Moralli S, Tarrado-Castellarnau M, Miranda A, Cascante M (2013). Targeting cell cycle regulation in cancer therapy. Pharmacol. Ther..

[26] Macintosh RL, Ryan KM (2013). Autophagy in tumour cell death. Semin. Cancer Biol..

[27] Galluzzi L (2017). Molecular definitions of autophagy and related processes. EMBO J..

[28] Vakifahmetoglu-Norberg H, Xia HG, Yuan J (2015). Pharmacologic agents targeting autophagy. J. Clin. Invest..

[29] Klionsky DJ (2008). Guidelines for the use and interpretation of assays for monitoring autophagy in higher eukaryotes. Autophagy.

[30] White E, Mehnert JM, Chan CS (2015). Autophagy, metabolism, and cancer. Clin. Cancer Res..

[31] White E (2015). The role for autophagy in cancer. J. Clin. Invest..

[32] Guo JY, Xia B, White E (2013). Autophagy-mediated tumor promotion. Cell.

[33] Lorin S, Hamai A, Mehrpour M, Codogno P (2013). Autophagy regulation and its role in cancer. Semin. Cancer Biol..

[34] Kenific CM, Debnath J (2015). Cellular and metabolic functions for autophagy in cancer cells. Trends Cell Biol..

[35] Luo M (2017). Gartanin induces cell cycle arrest and autophagy and suppresses migration involving PI3K/Akt/mTOR and MAPK signalling pathway in human glioma cells. J. Cell. Mol. Med..

[36] Pathania AS (2016). Interplay between cell cycle and autophagy induced by boswellic acid analog. Sci. Rep..

[37] Mathiassen, S. G., De Zio, D., & Cecconi, F. Autophagy and the cell cycle: a complex landscape. *Front. Oncol.*10.3389/fonc.2017.00051(2017). 10.3389/fonc.2017.00051 PMC5374984 28409123

[38] Levine B, Sinha S, Kroemer G (2008). Bcl-2 family members: dual regulators of apoptosis and autophagy. Autophagy.

[39] Siveen KS (2014). Targeting the STAT3 signaling pathway in cancer: role of synthetic and natural inhibitors. Biochim. Biophys. Acta.

[40] Wu L (2015). HIF-2α mediates hypoxia-induced LIF expression in human colorectal cancer cells. Oncotarget.

[41] Li X (2014). LIF promotes tumorigenesis and metastasis of breast cancer through the AKT-mTOR pathway. Oncotarget.

[42] Yu H (2014). LIF negatively regulates tumour-suppressor p53 through Stat3/ID1/MDM2 in colorectal cancers. Nat. Commun..

[43] Albrengues J (2014). LIF mediates proinvasive activation of stromal fibroblasts in cancer. Cell Rep..

[44] Wang J (2016). N-myc downstream-regulated gene 2 inhibits human cholangiocarcinoma progression and is regulated by leukemia inhibitory factor/MicroRNA-181c negative feedback pathway. Hepatology.

[45] Liu SC (2013). Leukemia inhibitory factor promotes nasopharyngeal carcinoma progression and radioresistance. J. Clin. Invest..

[46] Canel M (2017). Nuclear FAK and Runx1 cooperate to regulate IGFBP3, cell-cycle progression, and tumor growth. Cancer Res..

[47] Huang H (2016). Upregulation of SQSTM1/p62 contributes to nickel-induced malignant transformation of human bronchial epithelial cells. Autophagy.

[48] Yu H (2017). Lycorine promotes autophagy and apoptosis via TCRP1/Akt/mTOR axis inactivation in human hepatocellular carcinoma. Mol. Cancer Ther..

[49] Smith JJ (2010). Experimentally derived metastasis gene expression profile predicts recurrence and death in patients with colon cancer. Gastroenterology.

