# Supplementary material for: Magnolin promotes autophagy and cell cycle arrest via blocking LIF/Stat3/Mcl-1 axis in human colorectal cancers
Source: Cell Death Dis. 2018 Jun 13;9(6):702. doi: 10.1038/s41419-018-0660-4 (PMC5999973; doi:10.1038/s41419-018-0660-4)

Supplementary materials

For

Magnolin promotes autophagy and cell cycle arrest via blocking LIF/Stat3/Mcl-1 axis in human colorectal cancers

Haiyang Yu, Shuangshuang Yin, Shiyue Zhou, Yingying Shao, Jiachen Sun, Xu Pang, Lifeng Han, Yi Zhang, Xiumei Gao, Chengyun Jin, Yuling Qiu, Tao Wang

Figure Legend for Supplementary Data

Supplementary Figure 1. Magnolin promotes apoptosis and increases p27 expression in CRC cells. (**a,b**) cells were treated with indicated concentrations of magnolin for 48 h. (**a**) The percentage of apoptotic cells was determined by Annexin V staining. Data are shown as mean±S.D. (n = 3); *, P < 0.05; **, P < 0.01 compared with control (Student's t-test). (**b**) The protein levels of Cleaved Caspase 3 and PARP were detected by western blot assays. (**c**) p27 expressions in xenograft tumors were examined by IHC staining. Representative images were conducted as indicated. ***, P < 0.001; Scale bars, 50 μm. Data are shown as mean ± s.d. (n = 9).

Supplementary Figure 2. Magnolin promotes autophagy in CRC cells. (**a,b**) Quantitative analysis of protein expression in Figure 2a. Data are shown as mean±s.d. (n = 3); *, P < 0.05; **, P < 0.01 compared with control (Student's t-test). **(c-f)** Cells were treated with magnolin with or without CQ respectively. (**c**) Cells were transfected with a reporter plasmid (mRFP-GFP-LC3), followed by a confocal laser scanning microscope. Right, total number of endogenous LC3 puncta per cell. (**d**) The endogenous LC-3B puncta formation was measured by IF analysis. Right, quantitative analysis of autophagosomes. For **c** and **d**, data are shown as mean ± s.d. (n = 3); **, P < 0.01 compared with control; $^{##}$, P < 0.01 compared with cells treated with magnolin (Student's t-test). Scale bar, 20 μm. (**e,f**) The levels of LC-3B, p62, Cyclin D1 and p27 proteins were examined by western blot assays. (**g**) p62 and NBR1 expression in

xenograft tumors were determined by IHC staining. Representative images were conducted as indicated. ***, $P < 0.001$; Scale bar, 50 μm. For **g**, data are shown as mean ± s.d. ($n = 9$). All the western data shown are representative of at least three independent experiments.

Supplementary Figure 3. Inhibition of autophagy blocks magnolin-induced cell cycle arrest. (**a**,**b**) Cells were transfected with control siRNA or siRNA against LC-3B followed by magnolin treatment. The cell cycle distribution was determined by flow cytometer. Data are shown as mean ± s.d. ($n = 3$); **, $P < 0.01$ compared with control; $^{\#\#}$, $P < 0.01$ compared with control or si.control transfected cells treated with magnolin (Student's *t*-test). **(c-e**) Cells were transfected with control siRNA or siRNA against Atg3 followed by magnolin treatment. (**c,d**) Cell viability was determined using MTT assay. Data are shown as mean ± s.d. ($n = 3$); **, $P < 0.01$ compared with si.control transfected cells; $^{\#\#}$, $P < 0.01$ compared with si.control transfected cells treated with magnolin (Student's *t*-test). (**e**) The colongenicity of CRC cells were determined at indicated concentrations for 14 days.

Supplementary Figure 4. Mcl-1 overexpression suppresses magnolin-regulated autophagic flux and cell cycle arrest. (**a,b**) Quantitative analysis of LC3-II and LC3-I ratio in Figure 4c. Data are shown as mean ± s.d. ($n = 3$); **, $P < 0.01$ compared with vector control transfected cells; $^{\#\#}$, $P < 0.01$ compared with vector control transfected cells treated with magnolin (Student's *t*-test). (**c-f**) Cells were

transfected with Mcl-1 (Mcl-1 Vec) or empty vector (Control Vec) and followed by magnolin treatment. (**c**,**d**) Cells were transfected with a reporter plasmid (mRFP-GFP-LC3), followed by a confocal laser scanning microscope. Right, total number of endogenous LC3 puncta per cell. Scale bar, 20 μm. (**e,f**) The cell cycle distribution was determined by flow cytometer. Data are shown as mean ± s.d. ($n = 3$); **, $P < 0.01$ compared with vector control transfected cells; $^{\#\#}$, $P < 0.01$ compared with vector control transfected cells treated with magnolin (Student's *t*-test).

Supplementary Figure 5. knockdown of endogenous LIF by siRNA markedly decreased *Mcl-1* mRNA levels. (**a**) Cells were transfected with control siRNA or siRNA against LIF. The mRNA levels of Mcl-1 were determined by real-time PCR. **, $P < 0.01$ compared with siRNA control (Student's *t*-test). (**b**) Quantitative analysis of LC3-II and LC3-I ratio in Figure 4k. Data are shown as mean ± s.d. ($n = 3$); **, $P < 0.01$ compared with si.control.

Supplementary Figure 6. Full scans of western-blot data shown in Figure 1. Rectangles delimit cropped areas used in the indicated panels in Figure 1.

Supplementary Figure 7. Full scans of western-blot data shown in Figure 2. Rectangles delimit cropped areas used in the indicated panels in Figure 2.

Supplementary Figure 8. Full scans of western-blot data shown in Figure 3.

Rectangles delimit cropped areas used in the indicated panels in Figure 3.

Supplementary Figure 9. Full scans of western-blot data shown in Figure 4.

Rectangles delimit cropped areas used in the indicated panels in Figure 4.

Supplementary Figure 10. Full scans of western-blot data shown in Figure 5.

Rectangles delimit cropped areas used in the indicated panels in Figure 5.

Supplementary Figure 11. Full scans of western-blot data shown in Figure 6.

Rectangles delimit cropped areas used in the indicated panels in Figure 6.

Supplementary Figure 1

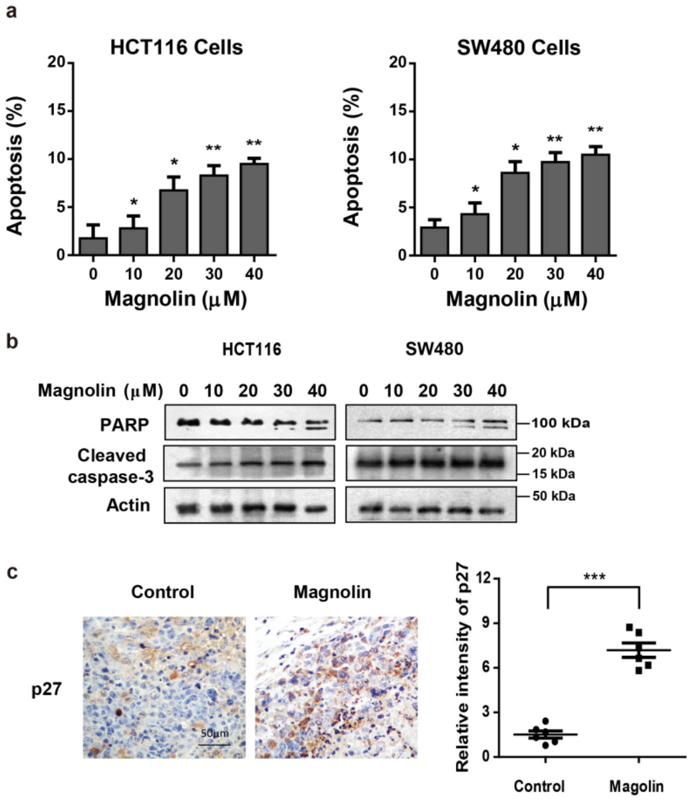

Supplementary Figure 2

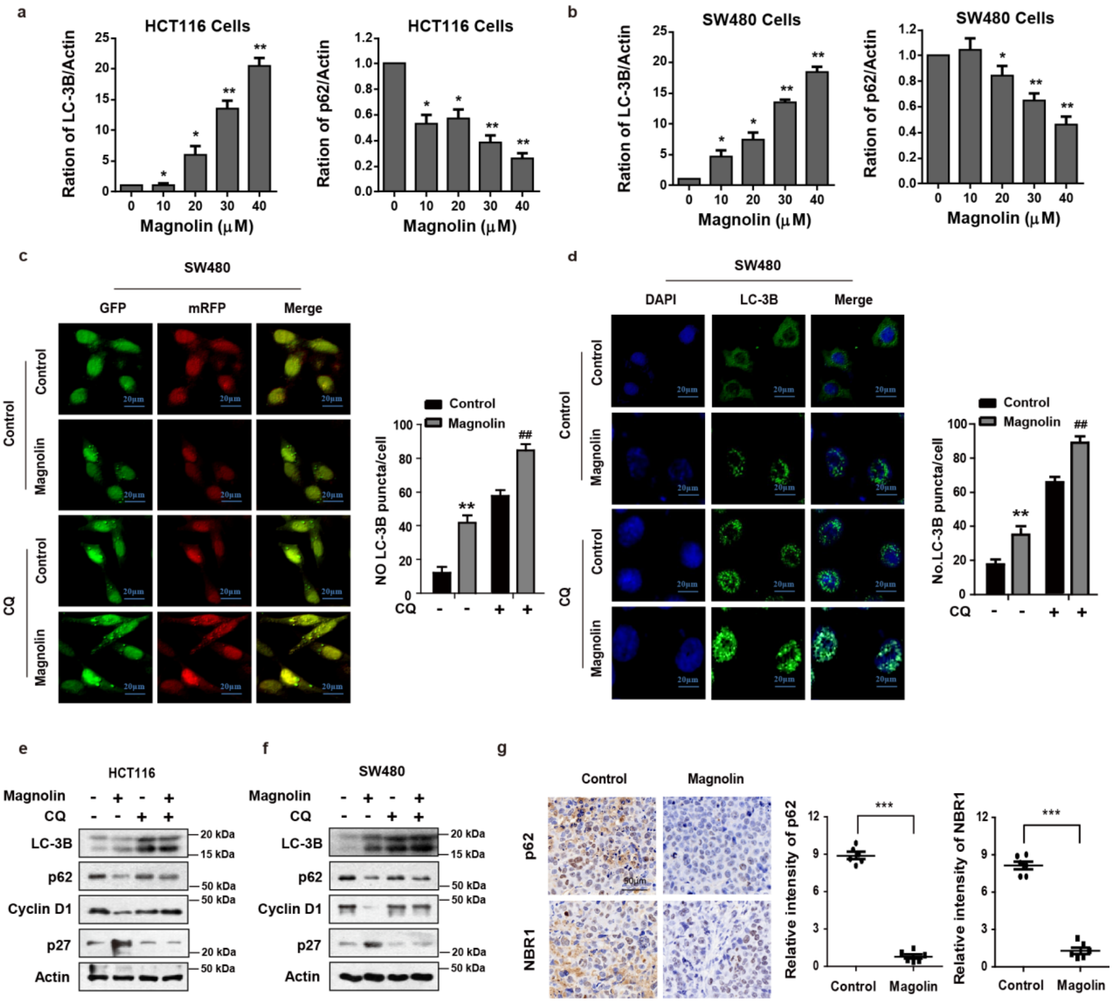

Supplementary Figure 3

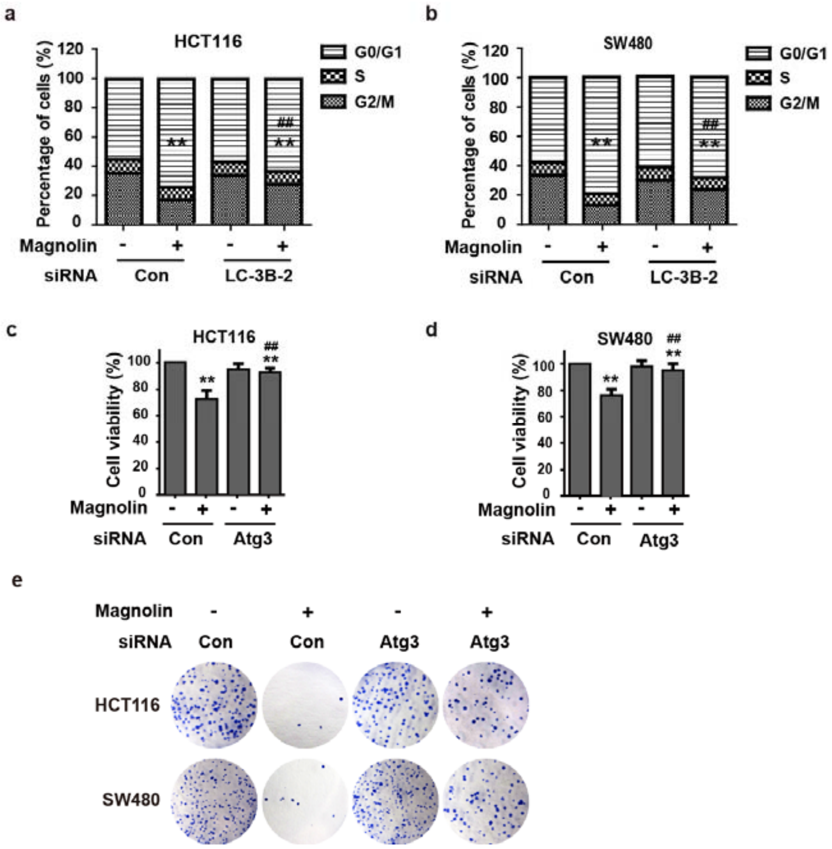

Supplementary Figure 4

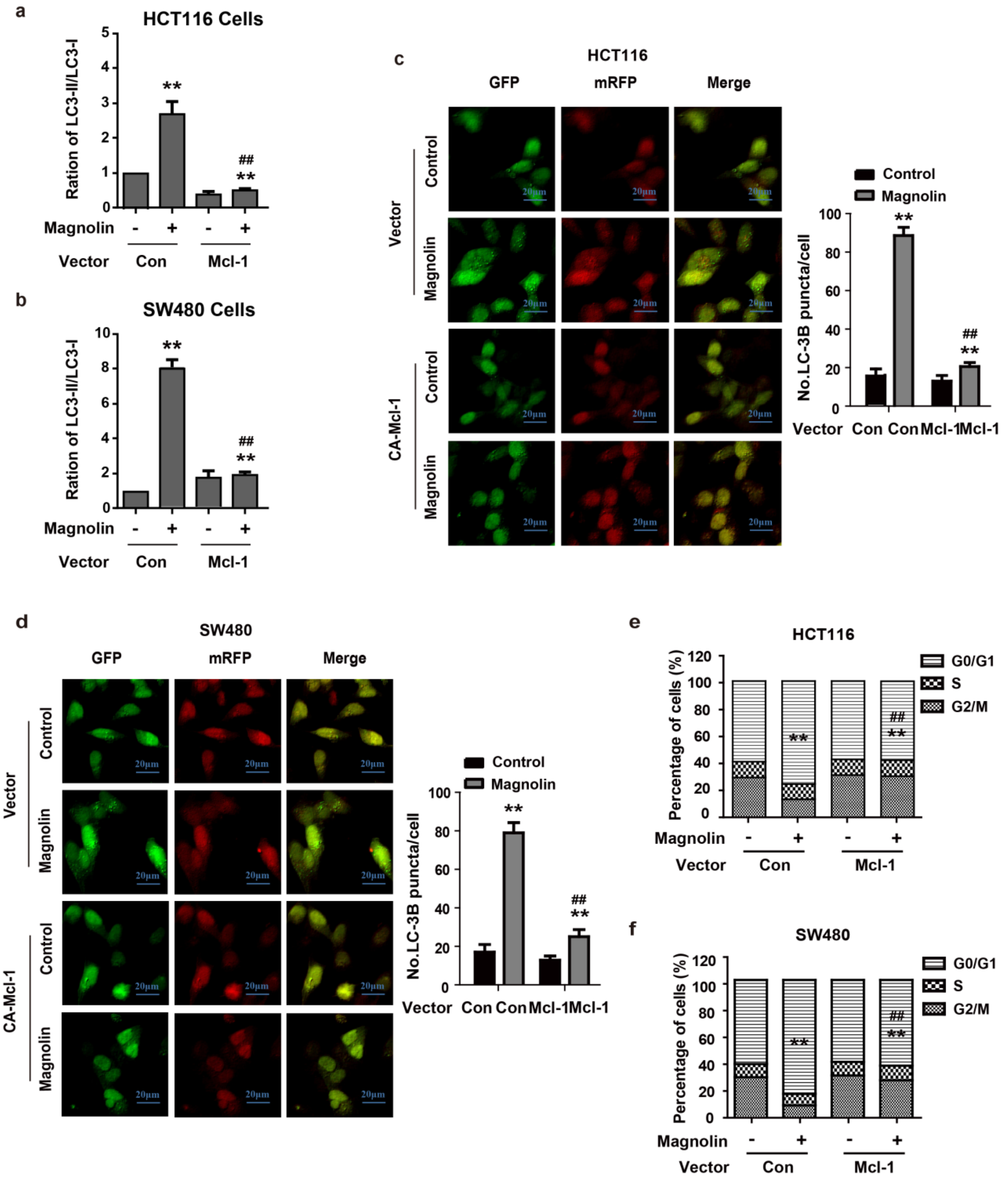

Supplementary Figure 5

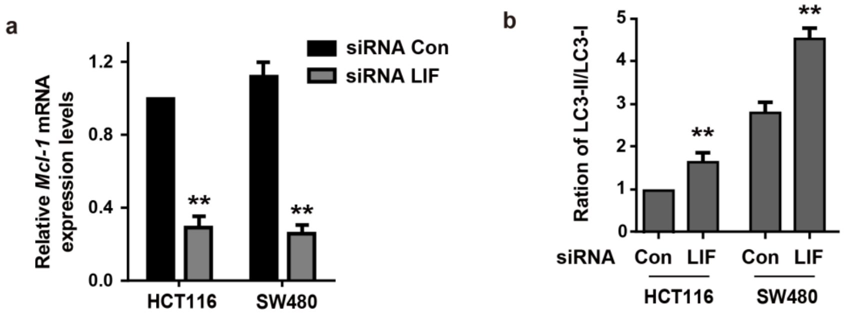

Supplementary Figure 6

Figure 1f

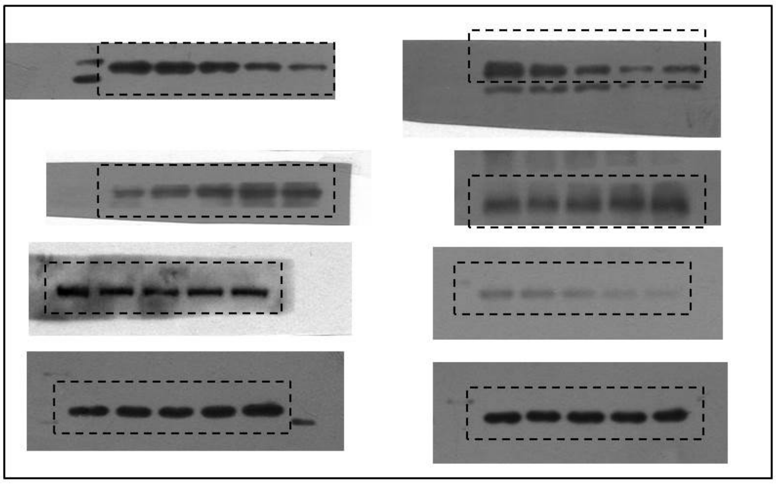

Figure 1g

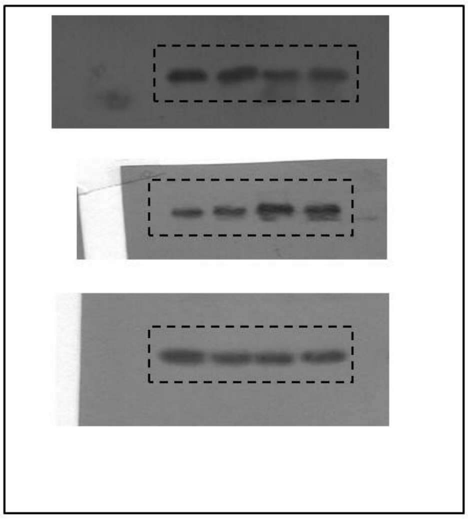

Supplementary Figure 7

Figure 2a

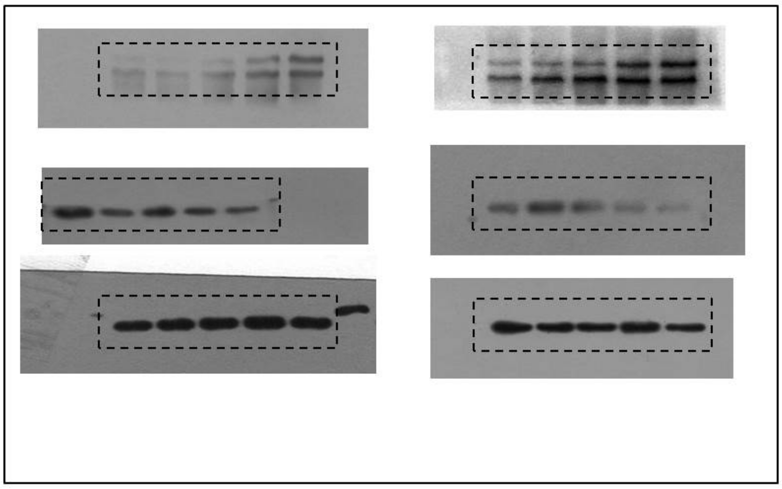

Figure 2e

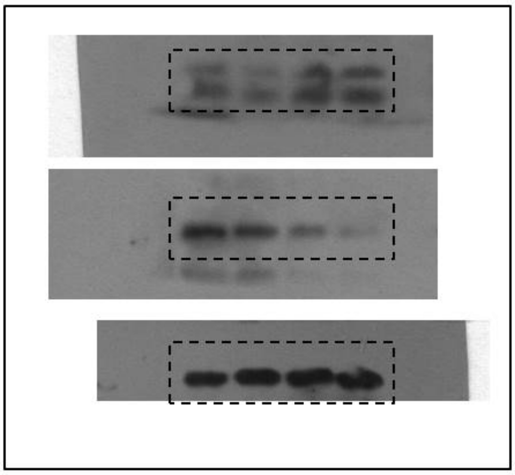

Supplementary Figure 8

Figure 3a

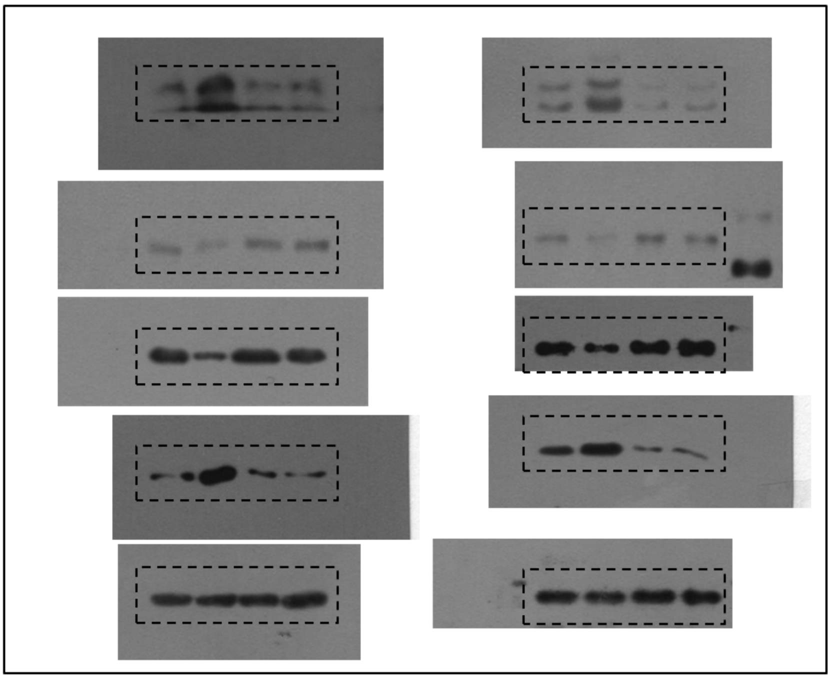

Figure 3c

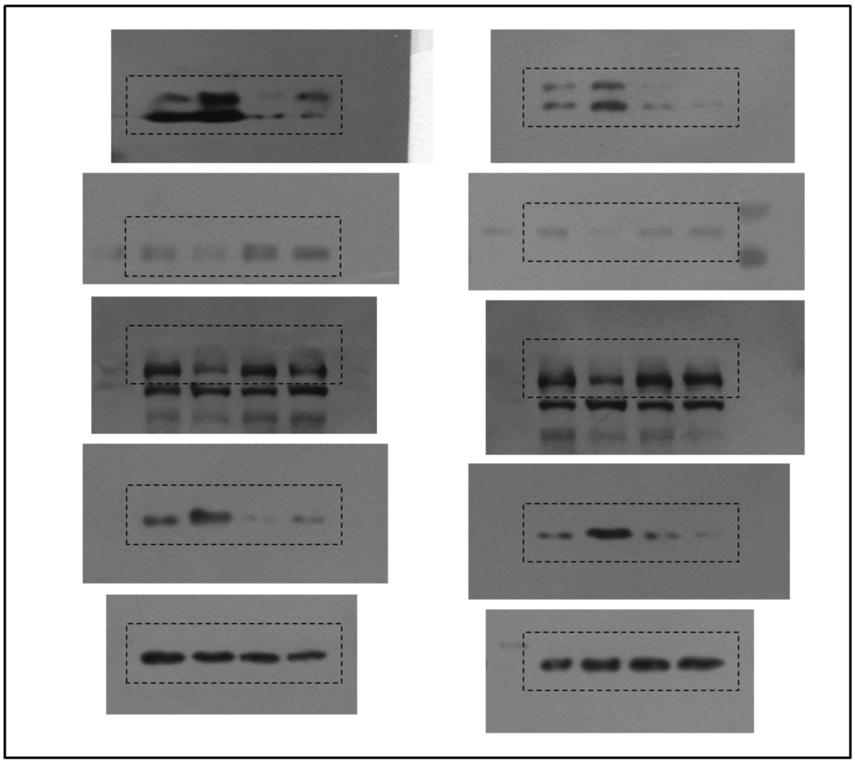

Supplementary Figure 9

Figure 4A

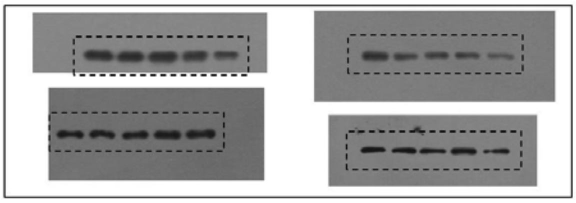

Figure 4C

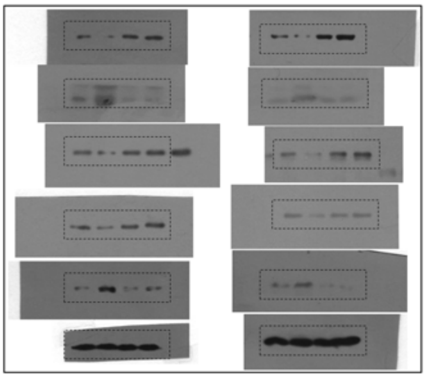

Figure 4D

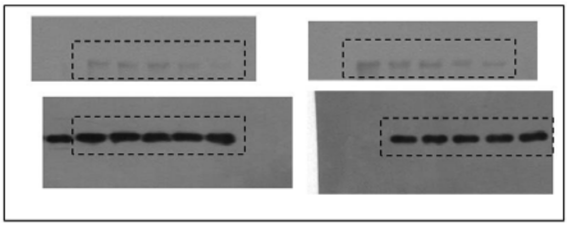

Figure 4E and F

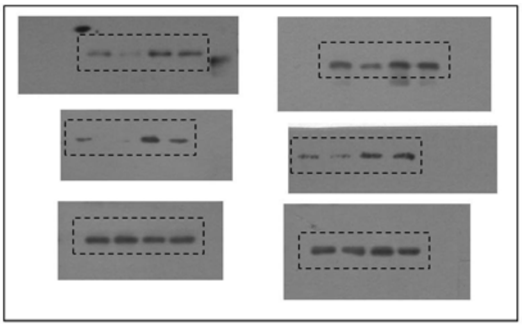

Figure 4J

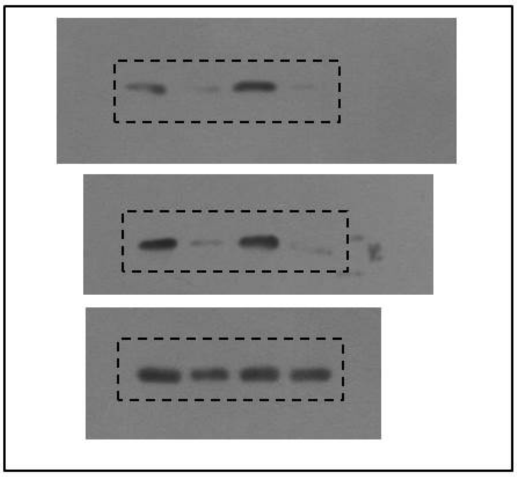

Figure 4K

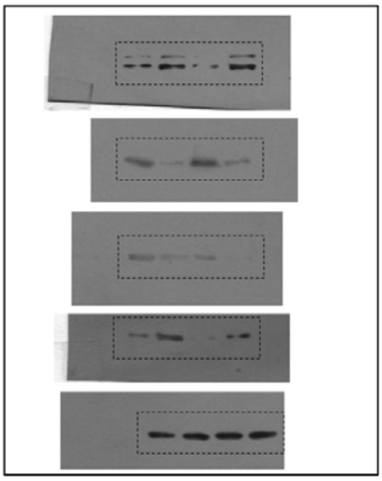

Supplementary Figure 10

Figure 5a

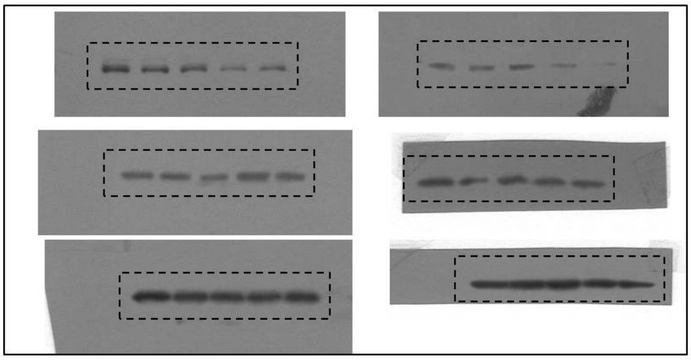

Figure 5b

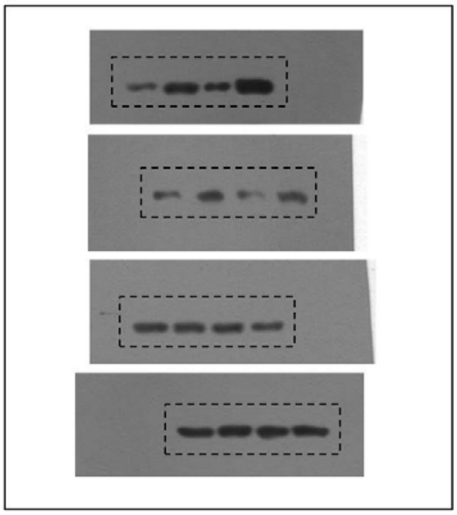

Figure 5c

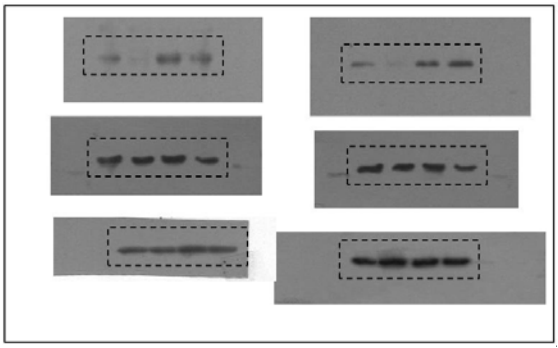

Figure 5d and e

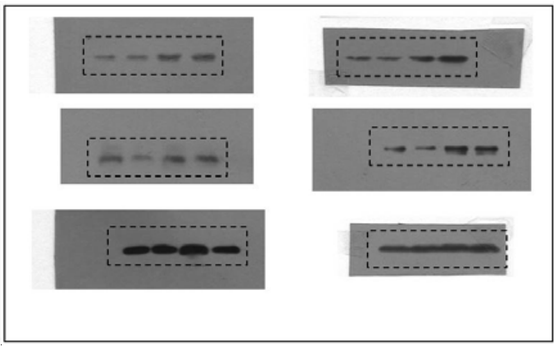

Figure 5f

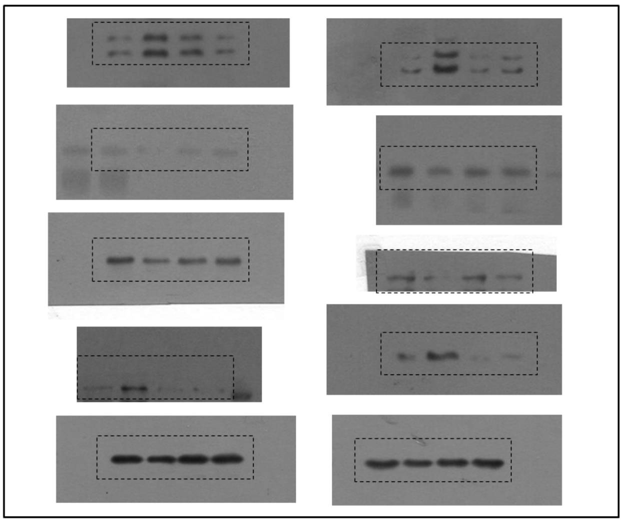

Supplementary Figure 11

Figure 6g

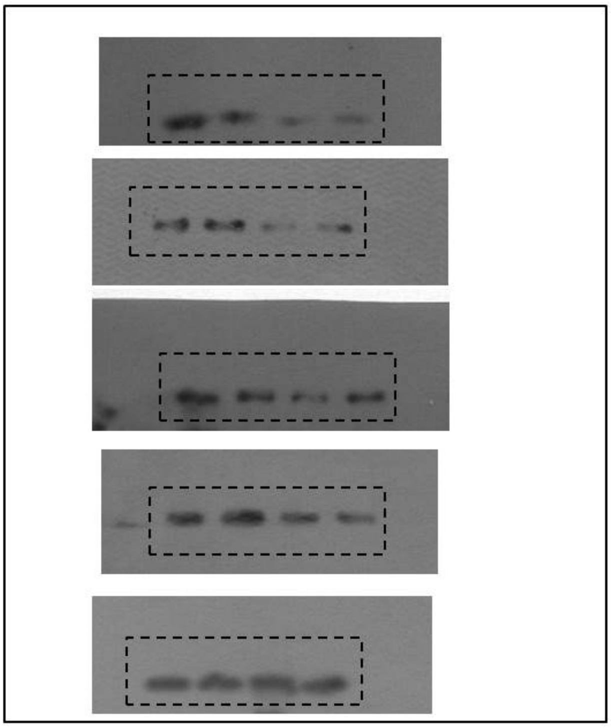

Supplement: Supplementary file 1 — Supplementary Data Cell Death and Disease [file 41419_2018_660_MOESM1_ESM.pdf]
